# ALDH Activity Selectively Defines an Enhanced Tumor-Initiating Cell Population Relative to CD133 Expression in Human Pancreatic Adenocarcinoma

**DOI:** 10.1371/journal.pone.0020636

**Published:** 2011-06-13

**Authors:** Michael P. Kim, Jason B. Fleming, Huamin Wang, James L. Abbruzzese, Woonyoung Choi, Scott Kopetz, David J. McConkey, Douglas B. Evans, Gary E. Gallick

**Affiliations:** 1 Department of Surgical Oncology, The University of Texas MD Anderson Cancer Center, Houston, Texas, United States of America; 2 Department of Pathology, The University of Texas MD Anderson Cancer Center, Houston, Texas, United States of America; 3 Department of Gastrointestinal Medical Oncology, The University of Texas MD Anderson Cancer Center, Houston, Texas, United States of America; 4 Department of Urology, The University of Texas MD Anderson Cancer Center, Houston, Texas, United States of America; 5 Department of Surgery, The Medical College of Wisconsin, Milwaukee, Wisconsin, United States of America; 6 Department of Genitourinary Medical Oncology, The University of Texas MD Anderson Cancer Center, Houston, Texas, United States of America; University of Hong Kong, Hong Kong

## Abstract

**Background:**

Multiple studies in recent years have identified highly tumorigenic populations of cells that drive tumor formation. These cancer stem cells (CSCs), or tumor-initiating cells (TICs), exhibit properties of normal stem cells and are associated with resistance to current therapies. As pancreatic adenocarcinoma is among the most resistant human cancers to chemo-radiation therapy, we sought to evaluate the presence of cell populations with tumor-initiating capacities in human pancreatic tumors. Understanding which pancreatic cancer cell populations possess tumor-initiating capabilities is critical to characterizing and understanding the biology of pancreatic CSCs towards therapeutic ends.

**Methodology/Principal Findings:**

We have isolated populations of cells with high ALDH activity (ALDH^high^) and/or CD133 cell surface expression from human xenograft tumors established from multiple patient tumors with pancreatic adenocarcinoma (direct xenograft tumors) and from the pancreatic cancer cell line L3.6pl. Through fluorescent activated cell sorting (FACs)-mediated enrichment and depletion of selected pancreatic cancer cell populations, we sought to discriminate the relative tumorigenicity of cell populations that express the pancreatic CSC markers CD133 and aldehyde dehydrogenase (ALDH). ALDH^high^ and ALDH^low^ cell populations were further examined for co-expression of CD44 and/or CD24. We demonstrate that unlike cell populations demonstrating low ALDH activity, as few as 100 cells enriched for high ALDH activity were capable of tumor formation, irrespective of CD133 expression. In direct xenograft tumors, the proportions of total tumor cells expressing ALDH and/or CD133 in xenograft tumors were unchanged through a minimum of two passages. We further demonstrate that ALDH expression among patients with pancreatic adenocarcinoma is heterogeneous, but the expression is constant in serial generations of individual direct xenograft tumors established from bulk human pancreatic tumors in NOD/SCID mice.

**Conclusions/Significance:**

We conclude that, in contrast to some previous studies, cell populations enriched for high ALDH activity alone are sufficient for efficient tumor-initiation with enhanced tumorigenic potential relative to CD133^+^ and ALDH^low^ cell populations in some direct xenograft tumors. Although cell populations enriched for CD133 expression may alone possess tumorigenic potential, they are significantly less tumorigenic than ALDH^high^ cell populations. ALDH^high^/CD44^+^/CD24^+^ or ALDH^low^/CD44^+^/CD24^+^ phenotypes do not appear to significantly contribute to tumor formation at low numbers of inoculated tumor cells. ALDH expression broadly varies among patients with pancreatic adenocarcinoma and the apparent expression is recapitulated in serial generations of direct xenograft tumors in NOD/SCID. We have thus identified a distinct population of TICs that should lead to identification of novel targets for pancreatic cancer therapy.

## Introduction

Pancreatic adenocarcinoma is the fourth leading cause of cancer mortality in the United States and is characterized by early metastasis and resistance to conventional therapies [1]. Overall prognosis for pancreatic cancer patients remains poor due largely to late diagnosis and our limited understanding of genetic and epigenetic factors contributing to disease progression and therapy resistance. In recent years, cancer stem cells (CSCs), also referred to as tumor-initiating cells (TICs), have been implicated in tumor formation, progression, and therapy-resistance in multiple solid-organ cancers, including pancreatic adenocarcinoma [2], [3], [4], [5], [6], [7], [8], [9], [10], [11], [12], [13], [14], [15]. As such, identifying the molecular markers that best discern pancreatic TIC populations may provide opportunities to characterize critical molecular pathways involved in tumorigenesis for targeted therapies [16].

To date, the identification of pancreatic cancer stem cells has been contingent upon the enrichment of TIC populations through marker-dependent cell selection. Using this strategy, high activity of the intracellular enzyme, aldehyde dehydrogenase (ALDH), and the cell surface markers CD133 and co-expressed CD44/CD24 have been shown individually shown to possess tumor-initiating properties in different studies [8], [13], [17]. Thus, whether pancreatic adenocarcinoma tumors arise from different CSC/progenitor populations enriched through selection of alternative cell surface/functional phenotypes (i.e. CD44/CD24, CD133, or ALDH) or common CSC/progenitor populations exist that expresses shared, “universal” TIC markers remains unclear.

ALDH is an intracellular enzyme involved in retinoic acid metabolism and its activity has been shown to enrich for normal and/or malignant stem cell populations in multiple organ systems including breast, colon, blood, and brain [12], [18], [19], [20].

Many studies of CSCs were initially performed in hematologic systems[21]. In these systems, further examination of ALDH^high^ cell populations for sub-populations also expressing CD133 or CD34 have identified common, primitive multi-lineage progenitor and stem cell populations [22], [23]. For example, Hess et al. demonstrated hematopoietic stem cell populations expressing both ALDH^high^ and CD133 possess a 10-fold greater potential for long-term bone marrow reconstitution relative to cells enriched for CD133 expression alone [22]. In pancreatic cancer, a recent study by Rasheed et al. demonstrated increased tumorigenic potential of ALDH^high^/CD44^+^/CD24^+^ and CD44^+^/CD24^+^ pancreatic cancer cell populations with ALDH expression correlating with a worse prognosis in early stage pancreatic cancer patients [17]. These authors found minimal overlap between ALDH and CD44^+^/CD24^+^ cell populations (<0.1%), suggesting the existence of at least two distinct tumor-initiating populations within human pancreatic tumors [17]. In contrast to the above study, Hermann et al. identified pancreatic CSCs by the cell surface expression of CD133 and determined that CD133 was expressed in approximately 1–2% of pancreatic cancer cells[8]. Although ALDH expression was not examined in this study, co-expression of CD44 and CD24 was observed in ∼0.1% of CD133^+^ CSCs, again suggesting the possibility that multiple, distinct tumor-initiating populations exist in pancreatic adenocarcinoma. Examination of cell populations common among both studies, specifically the tumor-initiating capabilities of purified ALDH, CD133 and “double positive” ALDH^high^ /CD133^+^ cell-populations, has not been performed previously. Further refinement of ALDH^high^ sub-populations based upon cell surface CD133 expression, similar to hematologic systems, may therefore assist in the identification of more tumorigenic cell populations.

Given the different conclusions reached in separate studies as to which, if any, single marker enriches for pancreatic TICs, we sought to determine whether high ALDH activity is a primary determinant of at least a subset of pancreatic TICs and the sufficiency of ALDH^high^ and ALDH^low^ cell populations for tumor-initiation when enriched or depleted of cell populations also expressing CD133. Herein, using a strict, direct xenograft tumor system and limiting dilutions of sorted human pancreatic cancer cells injected into NOD/SCID mice, we demonstrate that cell populations enriched for high ALDH activity alone fulfill the major criteria of a CSC and efficiently recapitulate the phenotype of the original tumor independent of CD133 cell surface expression.

## Results

### Pancreatic cancer cells contain ALDH^high^ and ALDH^low^ cell populations and heterogeneously express ALDH1

**Figure 1:**
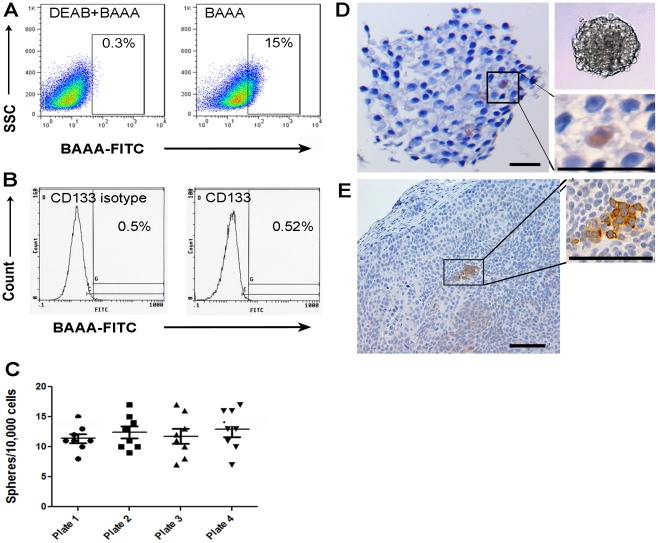
ALDH activity and expression in L3.6pl pancreatic cancer cells. (A) Representative example of L3.6pl cells stained with Aldefluor reagent with and without the DEAB inhibitor as described in Materials and Methods. Analysis by flow cytometry demonstrated high ALDH activity in approximately 16% of cells. (B) L3.6pl cells stained with directly conjugated CD133 antibodies failed to reveal a significant CD133^+^ population relative to isotype controls. (C) L3.6pl cells form spheres after plating on ultra-low attachment plates (average of 12 spheres/10,000 cells). (D) Cross sections of spheres were made and stained for ALDH1. A minority of cells contained within spheroids demonstrated strong ALDH1expression. (E) Staining for ALDH1 in sections of indirect xenograft tumors formed from L3.6pl cells in nude mice. Note small clusters of ALDH positive cells. Scale bar  = 20 µm (D), 50 µm (E).

We first evaluated ALDH activity and CD133 cell surface expression in a pancreatic cancer cell line (L3.6pl) previously used to enrich CSC populations through CD133 cell surface expression[8]. Flow cytometry demonstrated heterogeneous ALDH activity among L3.6pl cells with an average of 16.2% (median = 13.8%) ALDH^high^ cells relative to DEAB control samples, whereas CD133 expression was nearly undetectable (0.02%, median = 0.17) relative to isotype controls (Figure 1A, 1B). When plated in ultra-low attachment plates under conditions favoring the undifferentiated state, an average of 12 /10,000 L3.6pl cells (median = 11.5) possessed the ability to form spheroid structures and serial sections of paraffin-embedded spheres demonstrated the infrequent presence of cells expressing high levels of ALDH1 relative to all other cells (Figures 1C, 1D). Subcutaneously implanted L3.6pl cells in nude mice formed tumors with heterogeneous ALDH1 expression. ALDH1 expression was confined to small clusters of cells distributed throughout the tumors (Figure 1E). CD133 expression was not detected in indirect xenograft tumors formed from L3.6pl cells (data not shown).

**Figure 2:**
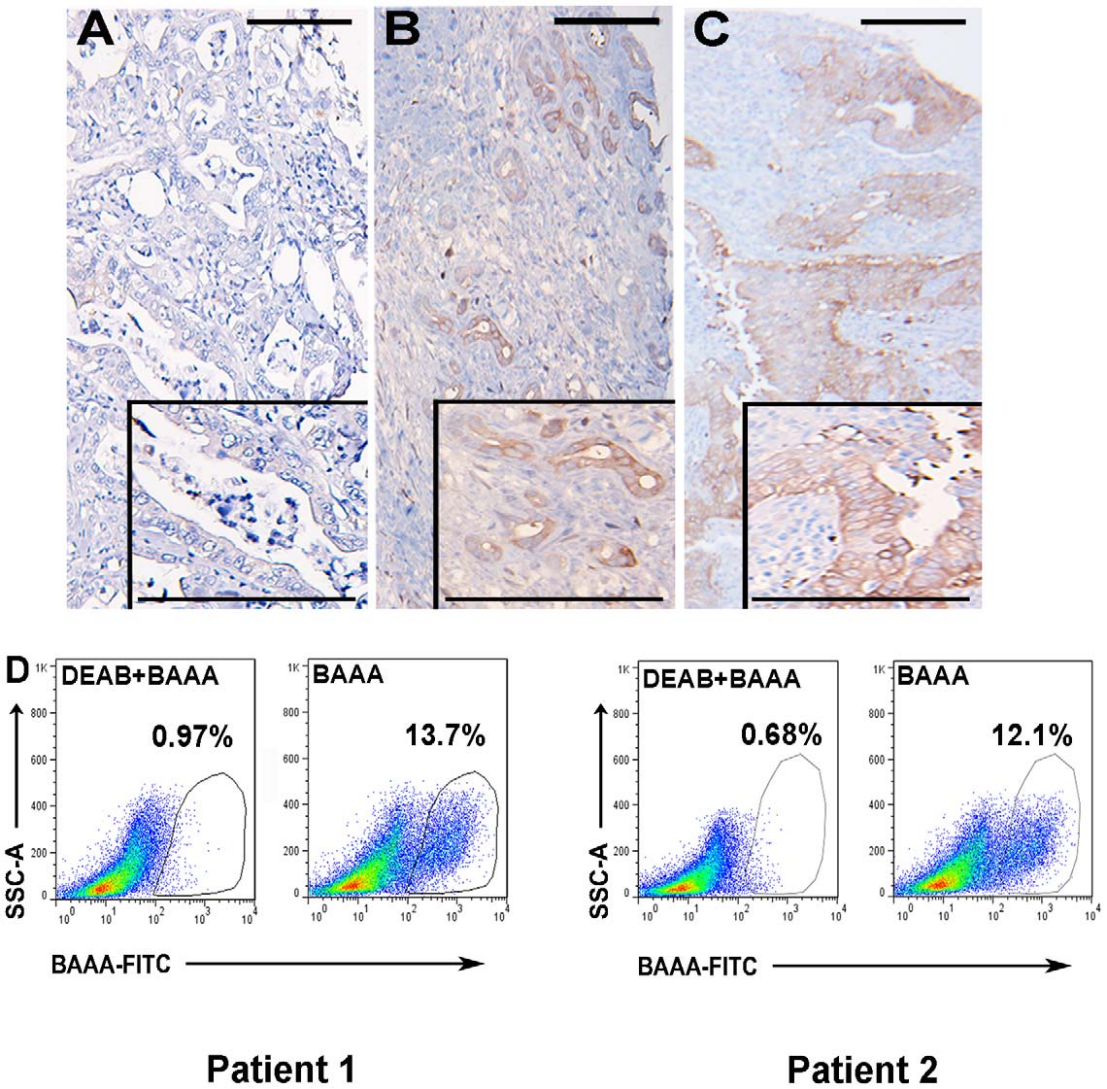
ALDH1 expression and activity in untreated primary pancreatic cancer specimens. Representative images of heterogeneous ALDH1 expression among patients with pancreatic cancer. Analysis of a TMA comprised of 106 untreated pancreatic tumors demonstrated (A) low ALDH1 expression in 22% (23/106) of examined patient specimens. (B) Moderate ALDH1 expression was detected in 26% (28/106) of examined patient specimens. (C) High ALDH1 expression was detected in 22% (23/106) of examined patient specimens. (D) Flow cytometry results of patient pancreatic adenocarcinoma tumors after digestion into a single cell suspension and stained with Aldefluor reagent, with and without DEAB inhibitor. Populations of cells with high ALDH activity relative to the overall cell population are easily distinguished. ALDH^high^ cells = 12.7% (Patient 1) and 11.4% (Patient 2) of all viable human cells. Scale bar  = 250 µm.

We next examined ALDH1 expression in primary human pancreatic adenocarcinoma tumors. To ascertain the intensity and location of tumor cells expressing ALDH1, we evaluated a tissue microarray (TMA) comprised of selected regions of pancreatic adenocarcinoma tumors with matched normal pancreas controls from 106 untreated patients. Immunohistochemical analysis of the specimens on the TMA demonstrated heterogeneous ALDH1 expression among patient specimens with ALDH expression beneath the level of detection in 32/106 (30%) samples. Low overall ALDH expression (corresponding with an expression score ≤2) was recorded in 23/106 (22%) patients (Figure 2A, see Materials and Methods for determination of quantitation); moderate overall ALDH expression was observed in 28/106 (26%) patient samples (Figure 2B, expression score >2 and ≤4); and high overall ALDH expression was observed in 23/106 (22%) patient samples (Figure 2C, expression score >4). These results demonstrate ALDH expression is heterogeneous among human pancreatic adenocarcinomas. ALDH expression was observed within luminal tumor cells and was exclusively confined to the cytoplasm. We next sought to confirm the presence of ALDH^high^ and ALDH^low^ cell populations in freshly resected pancreatic adenocarcinoma tumors by flow cytometry. We digested two patient tumors into single cell suspensions and stained with the Aldefluor® reagent, complete with DEAB-inhibited controls. Each digested patient tumor contained a single cell population demonstrating intensified ALDH activity relative to all other human tumor cells when analyzed by flow cytometry (Figure 2D, 2E). Collectively, these results demonstrate heterogeneous ALDH activity in individual human pancreatic tumors with only a minority of cells possessing ALDH^high^ activity.

### Histologic expression of ALDH1 is conserved in direct xenograft tumors

**Figure 3:**
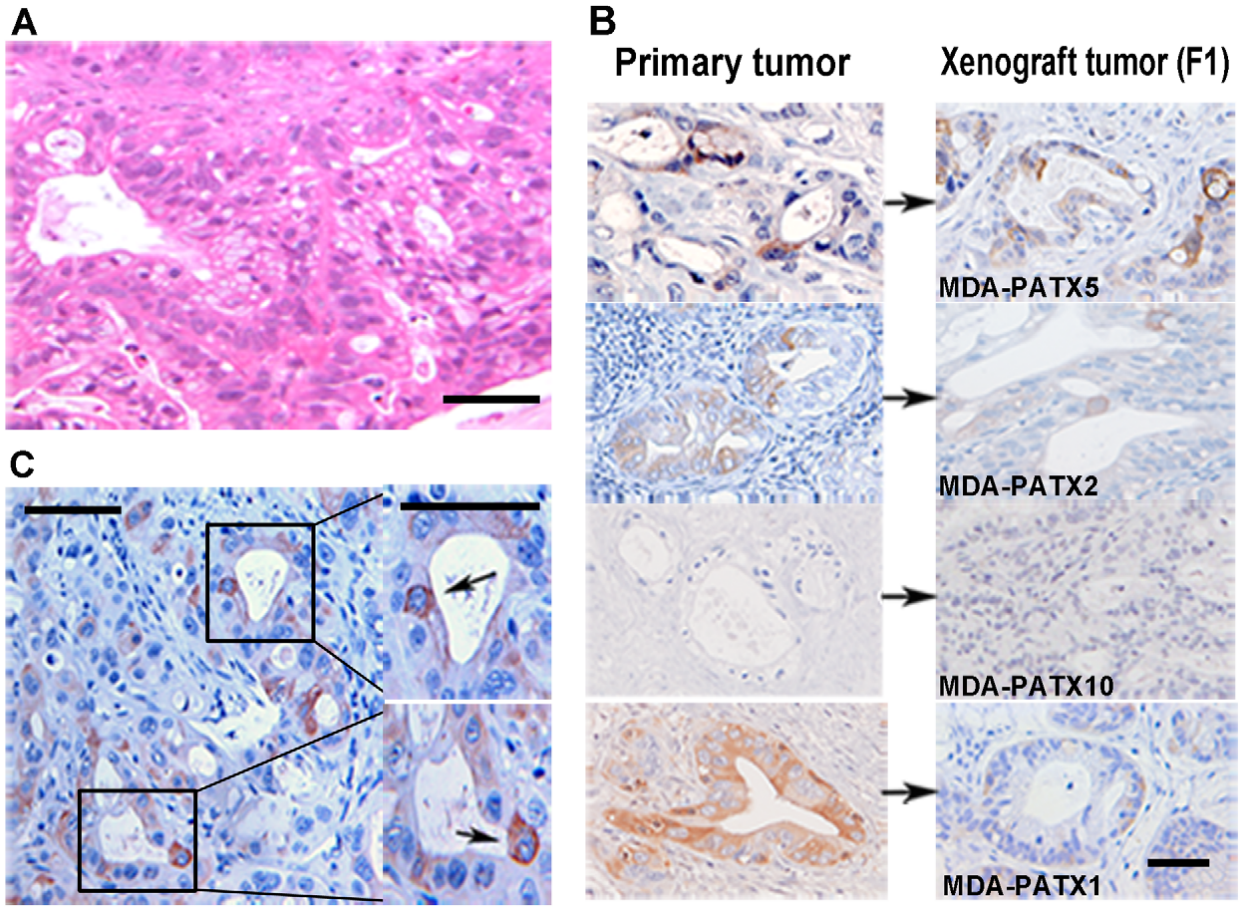
Histologic analysis of patient and direct xenograft tumors for expression of ALDH1. (A) Representative image demonstrating the histologic appearance of direct xenograft tumors established from freshly resected pancreatic tumors. Note tumor-gland formation and associated peri-tumoral stroma. (B) Comparison of ALDH1 expression in four different direct xenograft tumors to ALDH1 expression in original (parental) patient tumors. The pattern and location of ALDH1 expression is maintained during the xeno-transplantation process as reflected in derived xenograft tumors. An example of undetectable ALDH1 expression in both the patient tumor and derived direct xenograft is shown in the third panel from the top (MDA-PATX10). (C) Intra-tumoral heterogeneity of ALDH1 expression in direct xenograft tumors is readily identified as only a subset of luminal tumor cells demonstrate intense staining for ALDH1 relative to all other cells within tumor. Scale bar  = 125 µm (A), 180 µm (B), 125 µm (C).

To expand tumor specimens available for study, we established a panel of direct xenograft tumors to provide sufficient specimens for the study and isolation of pancreatic cancer cell populations. As we have reported previously, minced patient tumors were heterotopically implanted into NOD/SCID mice [24]. Direct xenograft tumors were established from surgical specimens resected from 11 different patients with pancreatic adenocarcinoma as confirmed by final pathologic diagnosis. Tumors were derived from patients already treated with neoadjuvant therapy (chemotherapy and radiation) and from patients who had not received any neoadjuvant therapy as shown in Table 1. Once engrafted, tumors were grown to a maximum diameter of 1.2 cm and surgically procured for histologic evaluation and serial implantation into additional generations of NOD/SCID mice. The overall histologic appearances of direct xenograft tumors (and at least two subsequent passages of these tumors) were nearly identical to the original patient tumors. As shown in the representative H&E image in Figure 3A, tumor-gland formation and associated peri-tumoral stroma were evident in all direct xenograft tumors.

**Table 1:** Analysis of ALDH activity and/or CD133 cell surface expression in different generations of direct pancreatic cancer xenograft tumors.

Patient/ treatment status	Xenograft generation	ALDH^low^/CD133^−^	ALDH^low^/CD133^+^	ALDH^high^/CD133^−^	ALDH^high^/CD133^+^
MDA-PATX5	F2	68.5	26.1	3.1	0.8
untreated	F2	49.32	45.6	2.6	1.32
	F3	43.8	46.8	5.4	2.54
	F3	41.3	49	6.9	2.7
	F3	-	-	4.6	-
	F3	59.2	37.8	1.8	0.9
	F4	-	-	4	-
	F4	-	15.5	2	-
	F4	76.7	21.6	2.1	0.4
	F4	86.9	12.6	0.4	0.1
	F4	88.4	9.7	1.8	0.1
	F4	87.9	8.3	3.6	0.1
	F4	69.5	27.8	2	0.7
	F4	73.6	23.7	2	0.6
	F4	-	6.8	2.3	-
MDA-PATX1	F2	43.1	8.5	42.9	5.5
untreated	F2	65.2	4.5	29.1	1.21
	F3	71.3	0.9	27.7	0.1
	F3	63.5	0.9	35.5	0.1
MDA-PATX13	F1	55.4	16.8	22.3	5.5
treated	F1	67.9	17.7	11.3	3.1
	F2	50.1	28.7	18.3	2.9
	F4	65.5	20.9	12.5	1.1
MDA-PATX2	F2	48.3	31.2	13.2	6.3
untreated	F3	37.9	29.2	26.5	6.4
	F3	20.8	40.8	26.4	12
MDA-PATX10	F1	97.9	0.1	2	0.01
treated	F2	-	-	15.2	-
MDA-PATX9	F1	74.6	2.7	22.6	0.1
treated					

We next compared the histologic presence and pattern of ALDH1-expressing cells in direct xenograft tumors relative to the parental (patient) tumors from which they were derived. Histologic evaluation of 12 direct xenografts established from 11 different patient tumors (one xenograft was derived from a lymph node metastasis from the same patient in which a xenograft was derived from a primary tumor) identified a subset cells that strongly express ALDH1 relative to all other tumor cells in all but one specimen (four examples are shown in Figure 3B). The patient specimen, MDA-PATX10, in which ALDH1 was undetectable by immunohistochemistry also did not express detectable ALDH1 in derived xenografts (Figure 3B), suggesting that the xeno-transplantation process did not affect ALDH1 expression. Again consistent with ALDH1 expression in human tumor specimens, intra-tumoral heterogeneity of ALDH1 expression in direct xenograft tumors was demonstrated through intense staining for ALDH1 in only a subset of luminal tumor cells relative to all other cells within tumor (Figure 3C). All of these characteristics were maintained in subsequent direct xenograft generations (data not shown).

### Comparison of ALDH^high^ and CD133^+^ cell populations in serial generations of NOD/SCID mice bearing xenografted human tumors

We next sought to identify and quantify overlapping and non-overlapping cell populations expressing putative pancreatic CSC markers in direct xenograft tumors by flow cytometry. Pancreatic tumors of NOD/SCID mice (F2–F4) underwent mechanical and enzymatic digestion into single cell suspensions and were subsequently stained with Aldefluor® reagent and directly conjugated anti-CD133 antibodies as described in Materials and Methods. After exclusion of debris and cells of mouse origin, viable human cells were analyzed by flow cytometry (see gating strategy, Figure S1).

**Figure 4:**
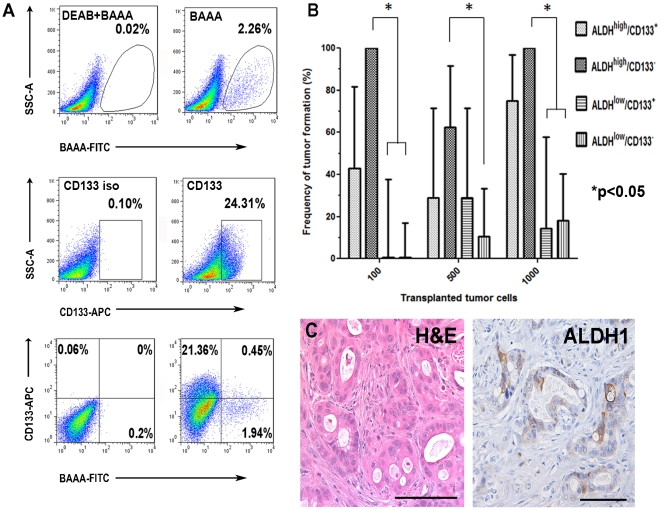
Isolation and quantitation of cells expressing high ALDH activity and/or cell surface CD133. (A) Representative images of digested tumors as analyzed by flow cytometry. After staining digested direct xenograft tumors with directly conjugated CD133 antibodies and the Aldefluor reagent (BAAA), ALDH^high^ and CD133^+^ cell populations were individually identified by flow cytometry, as described in Materials and Methods. Cell populations demonstrating high and low ALDH activity and/or CD133 expression were further purified using FACS. (B) Frequency of tumor formation after xeno-transplantation of cells expressing the specified markers at indicated numbers of inoculated cells. 95% confidence intervals are shown, *p<0.05. (C) Morpho-histologic appearance by H&E and ALDH expression in tumors initiated by ALDH^high^ cell populations. Tumor-gland formation, peri-tumoral stroma, and ALDH1 expression resembles that of parental, xenograft tumor. Scale bar  = 125 µm (C).

The results demonstrated ALDH^high^ and ALDH^low^ in all patient-derived xenografts examined, but the proportion of each varied widely (Figure 4A; Table 1). However, within a given patient xenograft lineage, the relative percentage of cells with high ALDH activity remained conserved through at least two passages in NOD/SCID mice as did the percentage of cell populations with low ALDH activity.

We next evaluated ALDH^high^ and ALDH^low^ cell populations for expression of CD133 on the cell surface and found the cell subset ALDH^high^ /CD133^+^ to be the most rarely expressed (range = 0.01–8.2% of all tumor cells) in all direct xenografts (Figure 4A; Table 1). The percent of ALDH^high^ /CD133^-^ tumor cells varied widely between xenograft tumors derived from different patients, with the vast majority of cells (60–90%) not expressing ALDH or CD133.

### Cell populations enriched for ALDH^high^ activity are more tumorigenic than ALDH^low^ and CD133^+^ cell populations

To determine which cell populations possessed the highest tumor-initiating potential, we injected purified ALDH^high^ /CD133^+^, ALDH^high^ /CD133^−^, ALDH^low^/CD133^+^ and ALDH^low^/CD133^−^ cells from pooled F2 generation tumors derived from MDA-PATX5 into the subcutaneous flank of NOD/SCID mice in limiting dilutions (100, 500, and 1000 cells), as described in Materials and Methods. All mice were monitored for the development of tumors for three months or sacrificed when tumors exceeded 1.2 cm.

ALDH^high^ /CD133^−^ cells demonstrated the highest incidence of tumor formation with tumors forming in all immunodeficient mice implanted with 100 cells (Figure 4B; Table 2). “Double positive,” ALDH^high^ /CD133^+^ cells were not significantly more tumorigenic than ALDH^high^ /CD133^−^ cells (p = .07). The incidence of tumor formation was much lower in ALDH^low^ cells, regardless of CD133 cell surface expression. Implantation of ALDH^low^/CD133^−^ cells resulted in a similar incidence of tumor formation relative to implanted ALDH^low^/CD133^+^ cell populations in all tested dilutions. Likewise, the incidence of tumor formation after implantation of 1000 ALDH^low^/CD133^+^ cells (14.2%) or 1000 ALDH^low^/CD133^−^ cells (18.2%) was significantly less than ALDH^high^ /CD133^+^ (75%) and ALDH^high^ /CD133^−^ (100%) cell populations (p = 0.04, p = 0.005 and, p = 0.007, p = 0.0002, respectively). At the lowest number of injected cells (100 cells), ALDH^high^ /CD133^−^ cell populations were significantly increased in incidence of tumor formation (100%) relative to ALDH^low^/CD133^+^ (0% tumor incidence) and ALDH^low^/CD133^−^ (0% tumor incidence) cell populations. Tumors initiated from ALDH^high^ populations recapitulated the histologic appearance of parental tumor complete with the pattern of ALDH1 expression (Figure 4C). Another large-scale tumor initiation experiment with identically sorted cell populations was performed using cells from pooled MDA-PATX1 xenograft tumors. In contrast to MDA-PATX5 xenograft tumors, MDA-PATX1 tumors possess low percent CD133^+^ cell populations (<10% tumor cells) and high ALDH^high^ percent cell populations (>25% tumor cells). From this population, ALDH^high^/CD133^−^ cells were also able to form tumors with high incidence, although overall tumor initiation was less in all sorted populations. Collectively, our findings demonstrate that ALDH^high^ /CD133^−^ pancreatic cancer cell populations purified from select direct xenograft tumors have enhanced tumor-initiating potential in NOD-SCID mice relative to ALDH^low^ cell populations.

**Table 2:** Incidence of tumor formation after xeno-transplantation of purified pancreatic cancer cell populations.

Markers	Number of transplanted cells		
	100	500	1000
**ALDH^high^/CD133^+^**	3/7	2/7	6/8
**ALDH^high^/CD133^−^**	6/6	5/8	7/7
**ALDH^low^/CD133^+^**	0/8	2/7	1/7
**ALDH^low^/CD133^−^**	0/21	2/19	4/22

*90-day incubation.

### ALDH^high^ cells produce tumors comprised of heterogeneous cell populations and recapitulate the morpho-histology observed in primary and direct xenograft tumors

**Figure 5:**
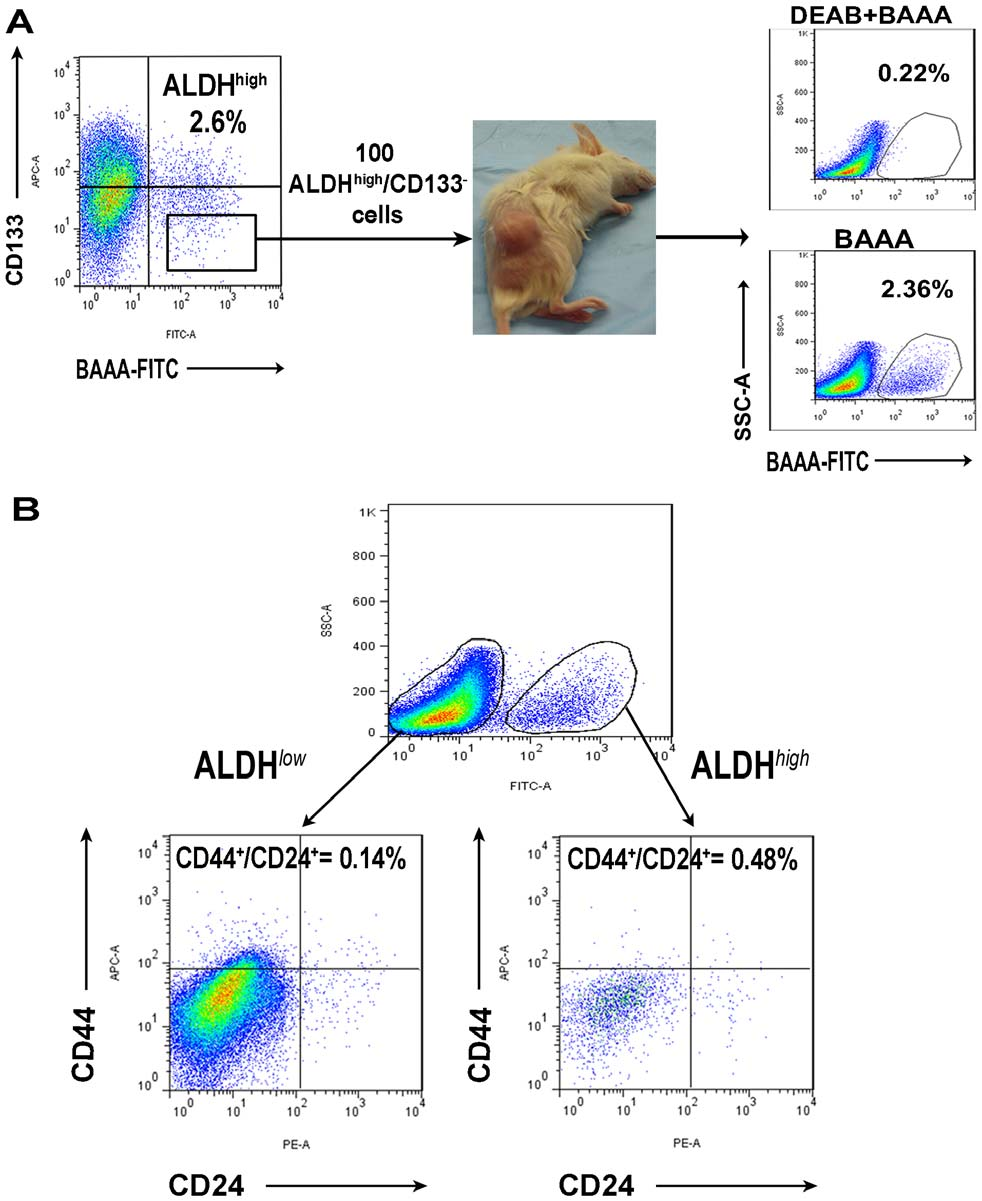
Analysis of direct xenograft tumors by flow cytometry after digestion into single cell suspensions. (A) Tumors initiated from 100 ALDH^high^/CD133^−^ cells were digested into single cell suspensions and analyzed by flow cytometry following staining with the Aldefluor reagent and directly conjugated CD133 antibodies. Both ALDH^high^ and ALDH^low^ cell populations were present in formed tumors in proportions (% cell population) similar to parental tumors. (B) Representative image of analysis of ALDH^high^ and ALDH^low^ cell populations for co-expression of CD44 and CD24 by flow cytometry. Small proportions of cells expressing CD44 and CD24 were present in both cell populations. ALDH^high^/CD44^+^/CD24^+^ cell populations comprised an average of only 0.015% of all viable, human pancreatic cancer cells and an average of 0.48% of all ALDH^high^ cells.

We next sought to determine whether tumors formed from ALDH^high^ cells resemble the histology of primary tumors and could generate the different cell populations observed in the parental direct xenograft tumors. Tumors initiated from 100 ALDH^high^/CD133^−^ cell populations were digested into single cell suspensions and subjected to analysis by flow cytometry as previously described. Tumors formed from ALDH^high^/CD133^−^ cell populations (derived from MDA-PATX5 direct xenograft tumors) produced both ALDH^high^ and ALDH^low^ cell subpopulations in similar proportions observed in parental tumors (Figure 5A). Tumors initiated from ALDH^high^ cells appeared histologically identical to parental tumors, demonstrating pseudogland formation and the same pattern of ALDH expression (Figure 4C). Taken together, these results demonstrate that tumors initiated from small numbers of ALDH^high^ cell populations recapitulate the histologic appearance of parental tumor and can reproduce both ALDH^high^ and ALDH^low^ cell subpopulations in tumors.

### ALDH^high^ and ALDH^low^ cell populations express CD44 and CD24

Previous reports demonstrated the enrichment of CSCs through the isolation of pancreatic cancer cells co-expressing the cell surface markers CD44 and CD24[13]. We therefore examined ALDH^high^ and ALDH^low^ cell populations for expression of cell surface CD44 and CD24 using flow cytometry (Figure 5B). ALDH^high^/CD44^+^/CD24^+^ cells comprised an average of 0.015% of all pancreatic cancer cells and ALDH^low^/CD44^+^/CD24^+^ cells comprised 0.11% of all tumor cells. Among ALDH^high^ cells, 0.48% (median = 0.38) co-expressed CD44 and CD24 and 0.12% (median = 0.14) of all ALDH^low^ cells were found to express CD44 and CD24 (Figure 5B). Although some increased expression of CD44 and CD24 was observed in ALDH^high^ populations relative to ALDH^low^ populations, the difference was not significantly significant (p = 0.09).

### Expression of ALDH and CD133 in direct xenografts derived from treated and untreated patient specimens

Multiple studies have shown that CSC populations become transiently enriched when tumors are subjected to chemotherapy and radiation [8]. Whether CSC populations are enriched in direct xenograft tumors established from patients already treated with chemotherapy and radiation therapy relative to tumors never exposed to such therapies remains unknown. We therefore compared ALDH activity and CD133 expression in xenograft tumors established from patient tumors treated with chemotherapy and radiation therapy relative to untreated specimens by flow cytometry. No general enrichment of either ALDH or CD133 expressing cell populations was observed in tumor specimens previously exposed to neoadjuvant therapy relative to treatment naïve tumor specimens (Table 1). However, because the percent cell populations of ALDH or CD133 expressing cells remains unknown in original patient tumors, we are unable to determine whether such cell populations become enriched or diluted in formed direct xenograft tumors.

## Discussion

We demonstrate that selection for cells with high ALDH activity from direct pancreatic cancer xenograft tumors enriches for TICs in the NOD/SCID xeno-transplantation model, with implantation of only 100 ALDH^high^ cells resulting in a 100 percent incidence of tumor formation. Our study represents the first prospective tumor-initiation study in this mouse model system examining the tumor-initiating capacity of pancreatic tumor cell populations based upon single and shared expression of the CSC markers ALDH and CD133. The first report of isolation of pancreatic cancer cells with tumor-initiating properties resulted from selection of cells for CD44, CD24, and ESA (epithelial-specific antigen) cell surface expression [13]. In contrast, Hermann et al. identified CD133 as a marker for tumor initiation in NOD/SCID mice [8]. A recent study by Rasheed et al. identified ALDH^high^ cells as possessing tumor-initiating properties with an incidence of tumor formation of 20 percent when 50–200 ALDH^high^ pancreatic tumor cells were implanted into NOD/SCID mice [17]. In the same study, ALDH^high^/CD44^+^/CD24^+^ cells initiated tumors in 36 percent of inoculated mice under identical conditions, but expression of CD133^+^ was not assessed in these cell populations, potentially important given the relatively low incidence of tumor formation (20–36 percent) in limiting dilutions of cells enriched for ALDH, CD44/24, or the combination thereof.

Given the different conclusions reached in separate studies as to which marker best enriches for pancreatic TICs, we focused on the relationship of ALDH and CD133 to establish the potential sufficiency of ALDH^high^ and/or CD133^+^ cell populations to efficiently initiate tumors. After demonstrating ALDH expression in a human pancreatic cancer cell line and in primary pancreatic cancer specimens through histologic analysis and flow cytometry, we next sought to further distinguish TICs through the purification of cell sub-populations based upon ALDH activity and/or CD133 cell surface expression from established direct xenograft tumors. In contrast to hematologic systems, we demonstrate that the “double positive,” ALDH^high^ /CD133^+^ phenotype is not significantly more tumorigenic than the ALDH^high^ /CD133^−^ phenotype in examined direct xenograft tumors with both cell phenotypes significantly more tumorigenic than ALDH^low^ cell populations. Tumors formed with 100 percent incidence from as few as 100 ALDH^high^ /CD133^−^ cells after xeno-transplantation in NOD/SCID mice, maintained the histologic appearance of the original tumor, and produced both ALDH^low^ and ALDH^high^ cell populations while re-establishing their relative prevalence (percent of all tumor cells) observed in parental tumors. A significantly reduced incidence of tumor formation was observed in cell populations enriched for CD133 alone relative to ALDH^high^ cell populations with minimal tumor formation observed in cell populations depleted of ALDH^high^ cells. Our results therefore implicate high ALDH activity as a more efficient and robust marker of pancreatic TIC populations than CD133 in the NOD/SCID mouse model system from the direct xenograft tumors we examined.

It should also be emphasized that we observed tumor formation from as few as 500 cells enriched for CD133 cell surface expression. Although not statistically different than CD133^−^ cell populations (p = 0.29), the tumor-initiating potential of cells enriched for CD133 is well documented in multiple studies from malignancies from various organ systems, including pancreatic cancer. A possible explanation for observed tumor formation from xeno-transplanted CD133^−^ and ALDH^low^ cell populations, particularly when more cells are inoculated, is contamination with more tumorigenic cells during high-volume cell sorting. Despite strict gating strategies under “gentle FACs” conditions, evaluation of sorted cell populations demonstrated >90% purity, reflecting inherent limitations in the fidelity of current cell sorting technologies. Our findings therefore do not strictly refute CD133 as a CSC marker, but merely identify a more tumorigenic cell population within the same tumors we analyzed, i.e. ALDH^high^ cell populations. These findings are in line with other studies that have observed tumor formation in limiting dilutions from cell phenotypes with less robust tumor-initiation capacity [10], [13].

As we have clarified the tumorigenic potential of distinct and overlapping populations of cells expressing ALDH and CD133, we also examined ALDH^high^ and ALDH^low^ cell populations for the co-expression of the cell surface markers CD44 and CD24. As discussed above, CD44 and CD24 have previously been identified as pancreatic CSC markers and represent a very small percent of the population of all tumor cells (0.1%) [13]. All examined xenograft tumors revealed very small overlap between CD44^+^/CD24^+^ and ALDH^high^ or ALDH^low^ cell populations, representing 0.015% and 0.11% respectively, of all viable, human tumor cells. We observed an approximate 4-fold enrichment of CD44^+^/CD24^+^ cells in purified ALDH^high^ cell populations relative to ALDH^low^ cell populations, a difference that was not statistically different due, in part, to limited sample size. Given the extreme rarity of ALDH^high^/CD44^+^/CD24^+^ cells in direct xenograft tumors (0.015% of all tumor cells), purification of ALDH^high^/CD44^+^/CD24^+^ cells was not technically feasible due to extended cell-sorting times and the mass quantity of tumor required to obtain even a small number of such cells. Moreover, given the low incidence of CD44^+^/CD24^+^ expression within ALDH^high^ cell populations (0.48% of ALDH^high^ cells), the presence of even one ALDH^high^/CD44^+^/CD24^+^ cell among 100 implanted ALDH^high^ cells is quite unlikely and, further still, would not likely account for the 100% incidence of tumor formation we observed within this purified cell sub-population. Taken together, these results provide further evidence that sorting for the ALDH^high^ cell population alone is sufficient to enrich for highly tumorigenic cell populations and that the low incidence of ALDH^high^/CD44^+^/CD24^+^ cells within purified ALDH^high^ cell populations implies that this cellular phenotype it is not a major contributor to tumor initiation. Our findings therefore suggest that high ALDH expression alone efficiently defines pancreatic TIC populations, a result consistent with other studies involving breast, ovarian, colon, and brain malignancies [2], [3], [4], [5], [12], [20].

A recently described complication in assessing the potential for tumor-initiation from sorted cells has been the strain of immune-deficient mouse used to study. For example, in melanoma, the incidence of tumor formation is very different in interleukin-2 receptor gamma chain (NSG) mice than in NOD-SCID mice [25]. However, Ishizawa et al. demonstrated recently that the frequency of tumor-initiating cells was higher in NSG than in NOD/SCID mice in only some tumors, but remained relatively similar in pancreatic and several other solid tumors where ALDH^high^ cells were examined for tumor initiation capacity [26]. Thus, in pancreatic cancer, the mouse strains used to date do not appear to affect the frequency of tumor initiation, further supporting the idea that CD24/CD44 cells in ALDH^high^ and ALDH^low^ populations are unlikely to contribute to tumor initiation in our studies.

Another important observation from our study is the inter-tumoral heterogeneity observed in regard to ALDH expression among patient and patient-derived xenografts. The relative percentage of ALDH^high^ cells was generally maintained in several generations of direct xenograft models despite some fluctuations within same-generation xenografts derived from common patient specimens. Such fluctuations may be technical in nature and due to varying degrees of xenograft tumor digestion, despite strict adherence to a functional protocol for the digestion of bulk tumor specimen. Analysis of direct xenograft tumors using flow cytometry yielded ALDH^high^ cell populations constituting 1–37% of all human tumor cells. The overall percent cell population of ALDH^high^ cells was furthermore re-established in tumors formed from as few as 100 ALDH^high^ cells, implying the presence of a persistent, fixed sub-population of TICs. Interestingly, tumors derived from patients who underwent neoadjuvant therapy were not enriched in either ALDH^high^ or CD133^+^ cells following xeno-transplantation. These findings are consistent with CSC theory which posits that small populations of CSCs may survive treatment and become transiently enriched, restoring tumor cell heterogeneity when more differentiated progeny are ultimately produced. It is therefore not surprising that once re-established in NOD/SCID mice, tumors derived from treated patients did not demonstrate any sustained enrichment in ALDH^high^ or CD133^+^ cell populations compared to tumors derived from untreated patient tumor specimens. Further investigations are required to determine whether putative CSC populations are enriched during chemo-radiation therapy in human pancreatic cancer patients and to isolate such populations for intense study towards therapeutic ends.

## Methods

### Ethics Statement

All surgically resected pancreatic tumors were collected after written patient consent and in accordance with the institutional review board-approved protocols of the University of Texas M.D. Anderson Cancer Center (protocol LAB07-0854). All animals are housed and maintained under guidelines established by the American Association of Laboratory and Animal Care and animal experiments are performed in accordance with NIH-Animal Care and Use Committee (ACUC) guidelines (protocols 09-07-10131 and 03-10-01431) after the University of Texas M.D. Anderson Cancer Center IRB approval.

### Cell lines and culture

The L3.6pl cell line was a gift from the laboratory of Dr. I.J. Fidler (The University of Texas M.D. Anderson Cancer Center) and was isolated from liver metastases after serial orthotopic implantation of the human pancreatic cancer cell line COLO357 in nude mice [27]. L3.6pl cells were maintained as a sub-confluent monolayer in MEM supplemented with 10% fetal bovine serum (Hyclone Laboratories, Waltham, MA), 2 mmol/L glutamine, and a penicillin-streptomycin mixture. All cells were incubated in 5% CO2 at 37°C. Cells were routinely screened for Mycoplasma and found to be Mycoplasma-free. For sphere formation assays, L3.6pl cells were counted with a haemocytometer and diluted to 10,000 cells/mL in stem cell media. Stem cell media was freshly made in DMEM/F12 (Hyclone Laboratories) supplemented with 1X B-27 supplement (Gibco, Carlsbad, CA), epidermal growth factor (EGF) (Invitrogen, Carlsbad, CA), and basic fibroblast growth factor (Invitrogen). One milliliter of cell suspension (10,000 cells/mL) was then plated into each well of 6-well ultra low-attachment plates (Corning, Lowell, MA) with an additional 0.5 ml of stem cell media added to each well every 2 days. After seven days, spheres were visualized and counted using light microscopy.

### Establishment of human pancreatic cancer xenografts

The establishment of direct heterotopic xenograft tumors in NOD/SCID mice has been described previously [24]. All NOD/SCID were purchased from the National Cancer Institute (Bethesda, Maryland). 4–6 week-old NOD/SCID mice were anesthetized with intra-peritoneal injections of a ketamine/xylazine cocktail and minced tumor fragments were implanted in subcutaneous flanks. Once grown to 1.2 cm in largest dimension, mice were sacrificed and tumor passaged into the subcutaneous flank of additional NOD/SCID mice.

### Immunohistochemistry

Paraffin-embedded human tumor samples, xenograft tumors, and cultured spheres were serially sectioned and deparaffinized in xylene and rehydrated in alcohol. Antigen retrieval was accomplished using citrate buffer pH6.0 and a conventional steamer followed by blocking in serum solution [28]. ALDH1 anti-human antibody (BD Biosciences, Franklin Lakes, NJ) was diluted 1∶100 and incubated with tissue sections overnight at 4°C. Pure CD133 anti-human antibody (Miltenyi Biotec, Germany) was diluted 1∶500 and incubated with tissue sections overnight at 4°C. Secondary antibodies used for immunohistochemistry were biotinylated goat anti-rabbit (Biocare Medical, Concord) diluted 1∶500 and developed using 3 3′ diaminobenzidine tetrahydrochloride (DAB). Slides were counterstained with hematoxylin and coverslipped with permount. Tissue samples were incubated with mouse IgG1 isotype controls (BD Biosciences, Franklin Lakes, NJ) and did not demonstrate any specific staining (Figure S2).

### Tissue microarray (TMA) evaluation of ALDH1 expression in human pancreatic ductal adenocarcinoma samples

A TMA composed of 106 human pancreatic ductal adenocarcinoma samples was prepared in the Department of Pathology at the University of Texas M.D. Anderson Cancer Center. For each tumor, two cores from representative areas of the tumor and one core from paired benign pancreatic tissue from the same patient were included. Both the intensity of antibody staining for ALDH1 and the gross percentage of ductal carcinoma cells demonstrating ALDH1 expression were assessed visually and graded. The intensity of ALDH1 staining was graded on a scale 0–3∶0 =  no staining; 1 =  weak staining; 2 =  moderate staining; 3 =  strong staining. Likewise, the percentage of tumor cells positive for ALDH1 expression was graded on a scale from 0–3∶0 =  no cells positive; 1 =  <10% positive cells; 2 = 10–25% positive cells; 3 =  >25% positive cells. Only ductal carcinoma cells were assessed and included in our analysis of pancreatic cancer specimens and matched controls (normal pancreas).

### Digestion of pancreatic tumors and flow cytometry

Xenograft tumors were dissected from host mice and minced with sterile scalpels in serum-free RPMI (Hyclone Laboratories) supplemented with type IV collagenase (200 units/mL, Worthington Biochemical, Lakewood, NJ) [24]. The cell solution was then sequentially passed through 70 µM and 40 µM nylon filters and incubated with RBC lysis buffer (eBioscience, San Diego, CA) for 10 minutes. The resulting single cells were then centrifuged and re-suspended in Aldefluor assay buffer (Stemcell Technologies, Vancouver, Canada) and counted with the aid of a haemocytometer. Cells were stained with the following directly conjugated monoclonal antibodies in the presence of FcR blocking reagent (Miltenyi Biotec): anti-mouse CD31-PE (phycoerythrin, Miltenyi Biotec), anti-mouse CD45-PE (BD Biosciences), anti-human CD133-APC (allophycocyanin, Miltenyi Biotec), anti-mouse H-2K^d^-PE (BD Biosciences), anti-human CD44-APC (BD Biosciences), and CD24-PE (BD Biosciences). The Aldefluor assay was performed per manufacturer's instructions (Stemcell Technologies) complete with DEAB controls. IgG isotype controls corresponding to each directly conjugated fluorophore were utilized to identify, quantify, and positively select desired cell populations. All analyses and cell sorting were performed on a BD FACSAria II (BD Biosciences) using FACSDiva (BD Biosciences) and FlowJo (Tree Star, Ashland, Oregon) software. Debris and cell clusters were excluded during side-scatter and forward-scatter analyses and cell viability determined using propidium iodide dye exclusion. Cells of mouse origin, including hematopoietic and endothelial progenitor cells, were removed through PE (phycoerythrin) exclusion. A 100-µm ceramic nozzle (BD Biosciences), sheath pressure of 25 pounds per square inch (PSI), and an acquisition rate of 1,000–5,000 events per second were used as conditions for cell sorting. Purity of sorted cell populations was routinely confirmed (>90%) through immediate flow cytometric analysis of pre-sorted cell populations.

### Xeno-transplantation of sorted human pancreatic cancer cells

Sorted cells were collected in sterile RPMI (Hyclone Laboratories) supplemented with 20% FBS chilled to 4°C. Sorted cells were then counted with a haemocytometer and serially diluted to desired concentrations in HBSS (BD Biosciences) containing 10% Matrigel (BD Biosciences) and kept on ice. NOD/SCID mice were anesthetized with a ketamine/xylazine cocktail and sorted cells were vortexed and immediately injected into the subcutaneous flanks with a 25G needle after the region was sterilized with a 70% ethanol solution. Mice were monitored until fully recovered from the effects of anesthesia and examined bi-weekly for tumor formation.

### Statistics

Differences in incidence of tumor formation among sorted cell populations were examined using the Fisher exact test. Differences in percent cell populations expressing CD44 and CD24 were evaluated using the Student's t-test.

## Supporting Information

Figure S1 **Strategy for analysis and isolation of viable, human pancreatic cancer cells from direct pancreatic cancer xenograft tumors.**
(JPG)

Figure S2 **Representative images of direct xenograft tumor sections stained with ALDH1A1 antibodies in parallel with appropriate IgG isotype controls.** Cytoplasmic staining was clearly visualized in tissue sections incubated with the ALDH1A1 antibody but not in tissue sections incubated with IgG controls.
(JPG)
