# Supplementary figures and images for: ALDH Activity Selectively Defines an Enhanced Tumor-Initiating Cell Population Relative to CD133 Expression in Human Pancreatic Adenocarcinoma
Source: PLoS One. 2011 Jun 13;6(6):e20636. doi: 10.1371/journal.pone.0020636 (PMC3113804; doi:10.1371/journal.pone.0020636)

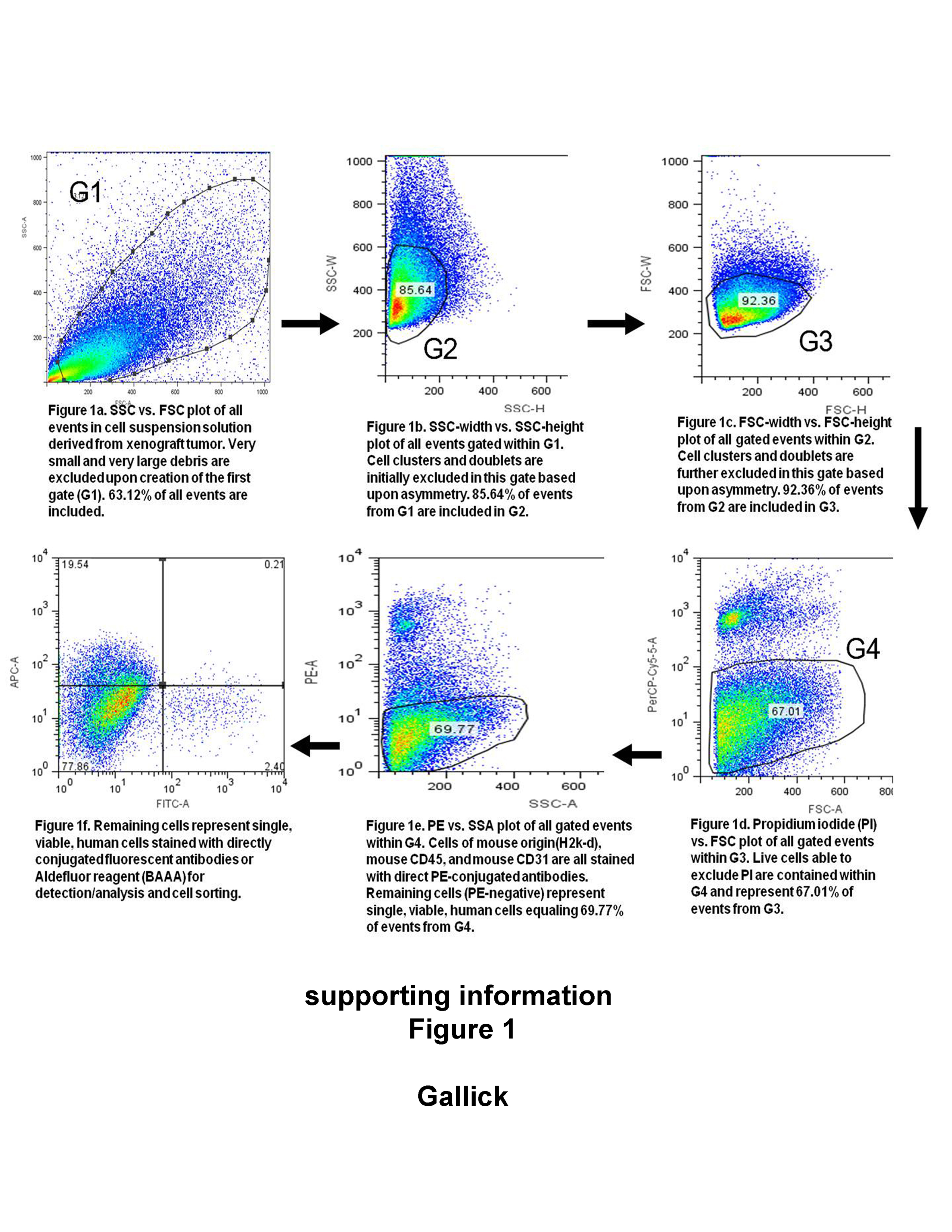

Supplement: Figure S1 — Strategy for analysis and isolation of viable, human pancreatic cancer cells from direct pancreatic cancer xenograft tumors. (JPG) [file pone.0020636.s001.jpg]

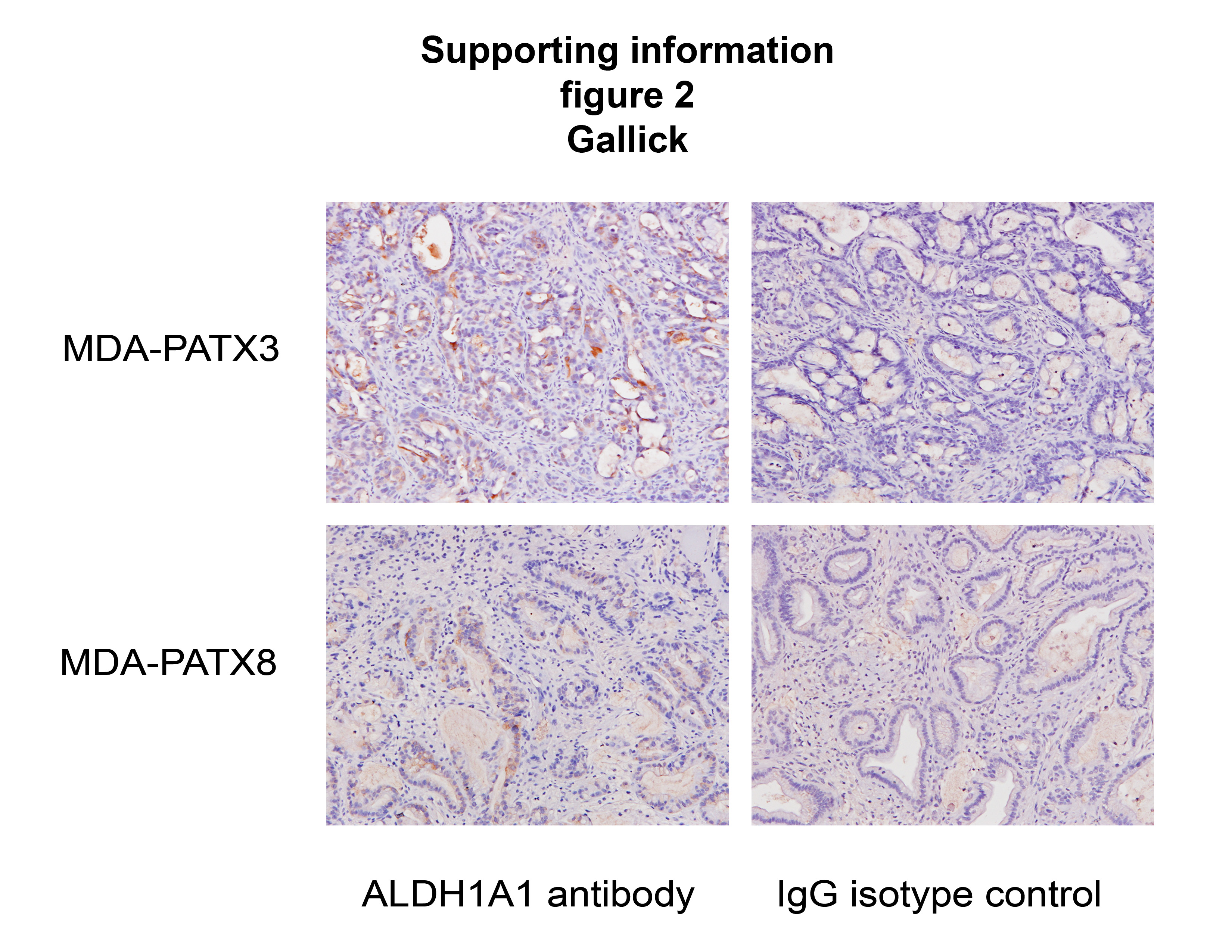

Supplement: Figure S2 — Representative images of direct xenograft tumor sections stained with ALDH1A1 antibodies in parallel with appropriate IgG isotype controls. Cytoplasmic staining was clearly visualized in tissue sections incubated with the ALDH1A1 antibody but not in tissue sections incubated with IgG controls. (JPG) [file pone.0020636.s002.jpg]
